# Clinical Value of Diffusion-Weighted Whole-Body Imaging with Background Body Signal Suppression (DWIBS) for Staging of Patients with Suspected Head and Neck Cancer

**DOI:** 10.3390/tomography8050210

**Published:** 2022-10-09

**Authors:** Andreas Schicho, Werner Habicher, Christina Wendl, Christian Stroszczynski, Quirin Strotzer, Marco Dollinger, Andreas G. Schreyer, Stephan Schleder

**Affiliations:** 1 Department of Radiology, University Medical Center Regensburg, 93053 Regensburg, Germany; 2 Department of Othorhinolaryngology, Merciful Brothers Hospital St. Elisabeth, 94315 Straubing, Germany; 3 Department of Diagnostic and Interventional Radiology, University Hospital Brandenburg, Brandenburg Medical School Theodor Fontane, 14770 Neuruppin, Germany; 4 Department of Diagnostic and Interventional Radiology, Merciful Brothers Hospital St. Elisabeth, 94315 Straubing, Germany

**Keywords:** DWIBS, CT, head and neck carcinoma, staging, metastases

## Abstract

(1) Background: To determine the importance of diffusion-weighted whole-body MRI with background body signal suppression (DWIBS) in the staging process of patients with suspected head and neck carcinomas. (2) Methods: A total of 30 patients (24 male, 6 female) with a median age of 67 years with clinically suspected head and neck carcinoma with pathologic cervical nodal swelling in ultrasound underwent the staging procedure with computed tomography (CT) and whole-body MRI including DWIBS. (3) Results: In a total of 9 patients, abnormalities in the routine work-up of pretherapeutic staging were found. Five cases of either secondary cancer or distant metastases were only visible in DWIBS, while being missed on CT. One diagnosis was only detectable in CT and not in DWIBS, whereas three diagnoses were recognizable in both modalities. (4) Conclusions: DWIBS in addition to a standard neck MRI in cervical lymphadenopathy suspicious for head and neck cancer yielded additional clinically relevant diagnoses in 17% of cases that would have been missed by current staging routine procedures. DWIBS offered a negative predictive value of 98.78% for ruling out distant metastases or secondary malignancies.

## 1. Introduction

Head and neck cancer has both a high mortality and morbidity due to impeding impairment of speaking, breathing, and swallowing. Worldwide, its incidence is rising with now about 14.500 new cases per year. In many cases, patients with head and neck cancer first present with cervical lymphadenopathy of unknown etiology, implicating a metastatic nodal stage in cases of malignoma detection [1]. 

In head and neck squamous cell carcinoma (HNSCC) patients, up to 20% present with a clinically identified distant spread of disease, while autopsy incidences have been reported to be up to 57% [2]. With proven distant metastases, palliative treatment remains the only option. Therefore, early detection of distant metastases is crucial to avoid curative treatment and associated side effects in a non-curable situation, and to direct patients with non-curable disease to the most promising palliative treatment [2].

Furthermore a high rate of patients with head and neck cancer is diagnosed with a secondary cancer in the pretherapeutic work-up, leading to a significant change in the therapy regimen [2]. 

Thus, in the situation of a cervical lymphadenopathy of suspected head and neck carcinomas, early whole body imaging would be of benefit for three reasons: first, to detect or rule out distant metastases in the assumption of a nodal-metastasized head and neck malignoma; second, to identify possible non head and neck primary tumors in the remaining cases, otherwise classified as carcinoma of unknown primary (CUP); third, to rule out secondary malignomas. 

The German S3-guideline for laryngeal cancer recommends contrast-enhanced computed tomography (CT) or contrast-enhanced magnetic resonance imaging (MRI) supplemental to the clinical and endoscopic findings for the local staging routine, leaning slightly to MRI due to its better soft tissue depiction. To rule out distant metastases, a thoracoabdominal CT scan is recommended [1,3,4].

### Whole-Body Imaging

In addition, for screening for distant metastases or in the diagnostic work-up of patients with a cervical CUP syndrome, 18 F-fluorodeoxyglucose positron emission tomography/computed tomography (18F-FDG-PET/CT) is used in select cases for whole-body imaging. The shortcomings of PET/CT, such as limited accuracy, time, cost, and nuclear invasivity, and the technical advancements in MRI on the other hand now open the possibility to offer whole-body-staging scans by MRI in a reasonable time without further patient preparation or technical prerequisites in a wider oncological spectrum. 

Due to the achievement of several technical upgrades, it is now clinically feasible to perform high-resolution whole-body magnetic resonance imaging protocols in a reasonable amount of time. In patients with head and neck carcinoma, whole-body magnetic resonance imaging already showed a promising role for the evaluation of distant metastases in such patients despite a spread in diagnostic accuracy compared to FDG-PET/CT [5,6,7]. 

In addition to conventional whole-body magnetic resonance imaging, diffusion-weighted imaging has shown potential. In order to deal with motion artifacts, Takahara et al. developed diffusion-weighted whole-body imaging with background body signal suppression (DWIBS). This sequence allows for the acquisition of diffusion-weighted imaging under free-breathing [2,8,9,10,11,12].

**Figure 1:**
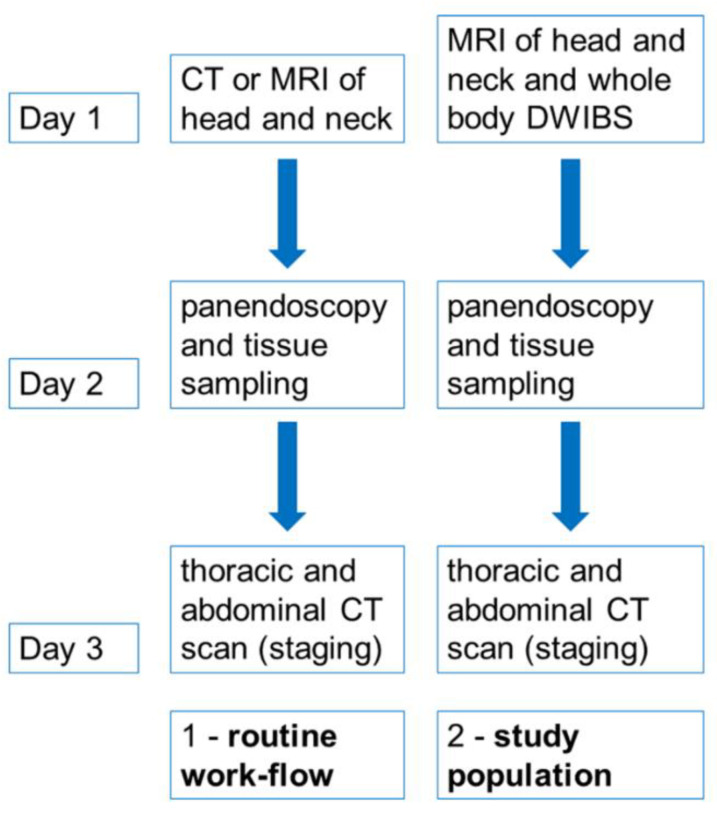
Routine diagnostic work-flow of patients with suspected head and neck cancer due to suspicious cervical lymphadenopathy compared to patients of our study population.

We here report on the clinical value of whole-body diffusion-weighted MR-imaging in patients with suspected head and neck cancer due to lymphadenopathy, who otherwise would only undergo local cervical MRI-based-staging and secondary CT- or PET/CT-staging if indicated. The standard clinical work-flow as recommended by the national guidelines compared to the study work-flow is presented in Figure 1.

## 2. Materials and Methods

### 2.1. Patients

The study was approved by the institutional ethics committee of the Medical Faculty of the University of Regensburg (No. 19-1492-104) and written informed consent was obtained from all patients. 

We enrolled 30 patients between December 2018 and November 2020 in our study. 

Patient inclusion criteria were: (1) Clinically suspected head and neck carcinoma in patients with pathologic cervical nodal swelling in ultrasound; (2) Staging with contrast-enhanced CT and MRI including whole-body DWIBS, from which clear images without apparent artifacts were obtained.

Patient exclusion criteria were: (1) history of head/neck chemoradiotherapy or other tumor treatment; (2) Contraindications for either contrast-enhanced staging CT (such as intolerance to iodine contrast agent) or MRI including whole-body DWIBS (such as severe claustrophobia, ferromagnetic foreign material).

After initial diagnostic work-up including histopathology, all patients and cases were discussed in a head and neck specialized tumor conference before initiation of treatment.

### 2.2. MRI Examination

MRI was performed using a 1.5 Tesla scanner (Ingenia, Philips Medical Systems DMC GmbH, Hamburg, Germany). Studies were performed with a MULTICOIL system (Philips Medical Systems DMC GmbH, Hamburg, Germany). No bowel preparation was performed before examination. The patients were placed in the supine position and positioned head-first on the table platform, ensuring that the body was covered from head to thigh. For the acquisition of the post-contrast-studies, intravenous injection of gadoteric acid (Dotarem, Guerbet Deutschland GmbH, Sulzbach/Taunus, Germany) was performed adapted to patient weight at a ratio of 0.2 mL/kg, varying in a dose of 15–20 mL.

Examinations consisted of (1) transversal T1-weighted images of the neck without fat saturation (turbospin echo; repetition time (TR) 590, echo time (TE) 10 ms, flip angle 90°) with a slice thickness of 4 mm, section gap of 1 mm, field of view (FOV) of 260 × 250 × 190 mm, matrix of 430 × 300 × 45 mm, and a scan time of about 2 min 50 s;

(2) transversal T2-weighted scans of the neck without fat saturation (turbospin echo; TR 9200 ms, TE 100 ms, flip angle 90°) with a slice thickness of 4 mm, section gap of 0 mm, FOV of 260 × 250 × 190 mm, matrix of 320 × 240 × 45 mm, and scan time of about 2 min 55 s;

(3) whole-body DWIBS in the axial plane with the following parameters: TR 10,030 ms, TE 70 ms, flip angle 90°, FOV 430 mm × 333 × 280 mm, matrix 144 × 110 × 70, 4 mm slice thickness, no section gap, b value 0 and 1000 s/mm 2, and a scan time of approximately 12 min;

(4) 3D-dixon-T1-weighted fat-saturated post-contrast study of the neck (TR 6 ms, TE 2 ms, flip angle 15°) with a FOV of 260 × 250 × 220 mm, matrix of 260 × 250 × 220 mm and scan time of about 3 min. with isotropic voxels of 1 mm and reformatted transversal, sagittal and coronal with a slice thickness of 3 mm, section gap of 0 mm.

### 2.3. CT Examination

Thoracic and abdominal scans were obtained with a 64-row MDCT scanner (Aquilion Prime, Toshiba Medical Systems Deutschland GmbH, Neuss, Germany). Enhanced CT images were obtained in all patients after the intravenous administration of iomeprol (Imeron, Bracco Imaging Deutschland GmbH, Konstanz, Germany) at a dose of 100 mL (350 mg of iodine per milliliter) and a rate of 3 mL/s. Thoracic scans were obtained during the arterial and abdominal scans during the portal venous phase that were determined with bolus tracking and automated triggering technology. Scan delay time in the arterial phase and portal venous phase were 15 and 50 s, respectively

Furthermore, an oral contrast agent was administered in all patients with 30 mL amidotrizioate (Peritrast-oral-GI, Dr. Franz Köhler Chemie GmbH, Bensheim, Germany) at a dose of 300 mg iodine per milliliter diluted in 1 L of drinking water one hour prior to the examination.

CT scans were performed in the supine position with the following parameters:

(1) thoracic scans: 120 kV; 40 mAs; transversal, sagittal and coronal reformations of 5 mm in thickness with a 5 mm gap and a collimation width of 0.5 mm;

(2) abdominal scans: 130 kV; 50 mAs; transversal, sagittal and coronal reformations of 5 mm in thickness with a 5 mm gap and a collimation width of 0.5 mm.

The transverse section data were additionally reconstructed with 1-mm-thick sections and then evaluated in the institutional PACS.

### 2.4. Image Analysis

All acquired images were analyzed in the PACS (Agfa Healthcare Deutschland GmbH, München, Germany) with dedicated diagnostic monitors (MDNC-2221, Barco Deutschland GmbH, Karlsruhe, Germany) prior to surgery and before the histopathologic results were known. Two radiologic residents with 10 and 20 years of experience, respectively, who were blind to the clinical and histopathological information interpreted each of the MR images independently. Differences in assessment were resolved by means of consensus.

### 2.5. Statistical Analysis

For data analysis, SPSS (IBM SPSS Statistics 28, IBM, Armonk, New York, USA) was used, into which an Excel file containing all the information collected was imported.

It was used to create all cross-tabulations and frequency tables in order to obtain a clear presentation of absolute and relative frequencies and to allow meaningful comparisons between the individual groups. SPSS was consequently also used to perform further descriptive analysis of the data set and illustrations. The McNemar test was used to test the research hypothesis. The statistical significance level was set as *p* < 0.05.

The intrinsic test characteristics (sensitivity and specificity) and the performance in the selected population (positive and negative predictive values) were calculated according to standard formulas using a 2 × 2 contingency table as follows: sensitivity (true positive rate, TPR) = TP/P; specificity (true negative rate, TNR) = TN/N; positive prediction value (PPV) = TP/(TP + FP); negative prediction value (NPV) = TN/(TN + FN); positive likelihood ratio (LR+) = TPR/FPR; negative likelihood ratio (LR−) = FNR/TNR; false negative rate (FNR) = 1 − TPR; where TP = true positive, P = positive, TN = true negative, N = negative, FP = false positive, FN = false negative, FPR = false positive rate, and FNR = false negative rate.

## 3. Results

### 3.1. Patient Characteristics

Of the 30 patients included, 24 (80%) were male, 6 (20%) were female. Median age was 67 years (male 65 years, women 73 years). The youngest patient was 34 years of age, the oldest was 89 years of age. A prevalence for relevant risk factors for head and neck carcinomas is shown in Table 1.

In all 30 patients included, a final malignant diagnosis was made. Six of the thirty malignancies (20%) were of non-head and -neck origin (leukemia (1), lymphoma (2), metastatic melanoma (1), metastatic bronchial carcinoma (2) after final histopathologic diagnosis), and five of the thirty malignancies were diagnosed as head and neck CUP (carcinoma of unknown primary) after complete diagnostic work-up (Table 2).

### 3.2. Findings of DWIBS

In a total of 9 patients, unexpected non-head and -neck diagnoses were found in the initial staging with neck MRI, DWIBS, and a thoraco-abdominal contrast-enhanced CT (Table 3) and were verified by histopathology.

One diagnosis of bronchial carcinoma was only detectable in computed tomography and not in DWIBS, whereas three diagnoses (bronchial carcinoma, breast lymphoma and liver metastases) were detectable in both modalities. Remarkably, five cases of either non-head and -neck primary or metastases were only visible in DWIBS (representing 16,7% of 30 patients), while being missed on computed tomography.

Overall, 5 out of 30 patients remained unclassified (CUP; 16,7%). The standard diagnostic work-up led to a correct diagnosis in 20 of 25 patients (80%), the improved diagnostic work-up with DWIBS diagnosed 24 of 25 patients (96%) correctly without CT and 25 of 25 patients (100%) with CT.

The results are statistically not significant (*p* = 0.219) but DWIBS was clearly superior to computed tomography only in the diagnostic work up of head and neck carcinoma.

The measures of diagnostic accuracy were calculated as follows, assuming the aggregate of both tests (DWIBS and CT) to correctly reflect the presence or absence of diseases assessable by imaging.

CT had a sensitivity of 44.4% (CI95 13.7–78.8%), a negative predictive value of 94.2% (CI95 90.0–96.7%), and a negative diagnostic likelihood ratio of 0.556 (CI95 0.31–0.99) for finding the primary malignancy.

DWIBS had a sensitivity of 88.89% (CI95 51.75–99.72%), a negative predictive value of 98,78% (CI95 92,73–99.81%), and a negative diagnostic likelihood ratio of 0.11 (CI95 0.018–0.705) for finding the primary malignancy.

### 3.3. Patients’ Further Clinical Course

All therapeutic decisions made for the 30 patients included are based on an institutional multidisciplinary tumor conference consensus and can be found in Table 4.

## 4. Discussion

In patients presenting with cervical lymphadenopathy suspected of head and neck cancer, diagnostic work-up and staging needs to be reliable and efficient. Distant metastases need to be identified before treatment is initiated. The incidence of synchronous second cancer or distant metastases in carcinomas of the head and neck region varies between 4% and 33%, depending on the size of the primary tumor with stages of T3 and T4 and patients with lymph node involvement being particularly frequently affected [13,14,15]. For example, a significantly higher rate of a secondary bronchial carcinoma was demonstrated in patients with an increase in T stage by Kaanders et al. in 2002 [16].

Depending on the results of initial staging, optimized and customized therapy recommendation can be made. The curative intended therapy is amenable only for patients with locoregionally confined disease. By standard, staging of thorax and abdomen is performed by contrast-enhanced computed tomography, ultrasound, and/or chest X-ray [2].

We here tested the diagnostic performance of a whole-body diffusion-weighted imaging sequence added to the neck MRI as a possible substitute of, or addition to, the CT.

In our study, five diagnoses were made by the DWIBS sequence that would otherwise have been missed by CT. Only in one case, a bronchial carcinoma, DWIBS-staging was inferior to CT. In four cases, both DWIBS and CT showed the pathology. Noji et al. reported a sensitivity and specificity of 93.8% and 73.3% for qualitative scoring of 18F-FDG-PET/CT in patients with head and neck cancer of unknown primary. They found no added clinical value through diffusion-weighted imaging. We here found a negative predictive value of 98.78% for DWIBS in the staging for distant metastases or secondary malignancies. DWIBS has the advantage of being easy to add to any head-and-neck MRI without the need of scheduling a tracer-dependent, time and cost consuming second modality. Highly specialized diagnostic techniques such as PET-CT or PET-MRI should only be used in non-conclusive cases [2,17,18]. Fine-needle aspiration increases the accuracy of diagnosis according to studies but is an invasive measure, as is trans-oral robotic surgery or trans-oral laser microsurgery for tonsillectomy. Further studies should elaborate not only on the clinical value of different diagnostic strategies, but on diagnostic’s morbidity, individual, and socio-economic burden as well, while whole-body MRI is increasingly used as the staging method [18,19].

Based on the here presented results, a possible diagnostic routine for patients with cervical lymphadenopathy suspicious for metastases of an unknown primary should contain a neck MRI with DWIBS and a two plane chest X-ray to compensate for the low sensitivity of MRI in the detection of pulmonary nodules. This approach combines the advantages of being fast, low cost, and low radiation while having a high-sensitivity and -specificity.

The main limitation of the study is the low number of patients enrolled and the single-center design. Both can be addressed in further follow-up studies.

Furthermore, the inclusion criteria of clinically suspected head and neck carcinoma in patients with cervical lymphadenopathy may lead to the bias of advanced tumor disease with a higher rate of findings in staging examinations.

## 5. Conclusions

Whole-body diffusion-weighted imaging in addition to a neck MRI in cervical lymphadenopathy suspicious for head and neck cancer yields additional clinically relevant diagnoses in 17% of cases, that would have been missed by conventional neck MRI and CT alone. Regarding the rule-out of distant metastases or secondary malignancies, DWIBS has a high negative predictive value of 98.78%. Larger studies should now verify the value of a modified diagnostic work-up including DWIBS for patients presenting with cervical lymphadenopathy.

## Figures and Tables

**Figure 2:**
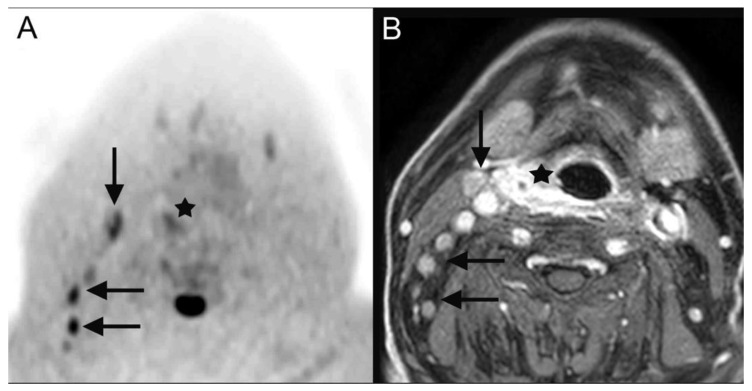
DWIBS (**A**) as well as contrast-enhanced fat-saturated T1-weighted MR images (**B**) depict the primary cancer (**A**,**B**: black star) lesion as well as the nodal metastasis (**A**,**B**: black arrows) in a 70-year-old male patient who was diagnosed with a right-sided hypopharyngeal carcinoma (cT2 cN2b cM0).

**Figure 3:**
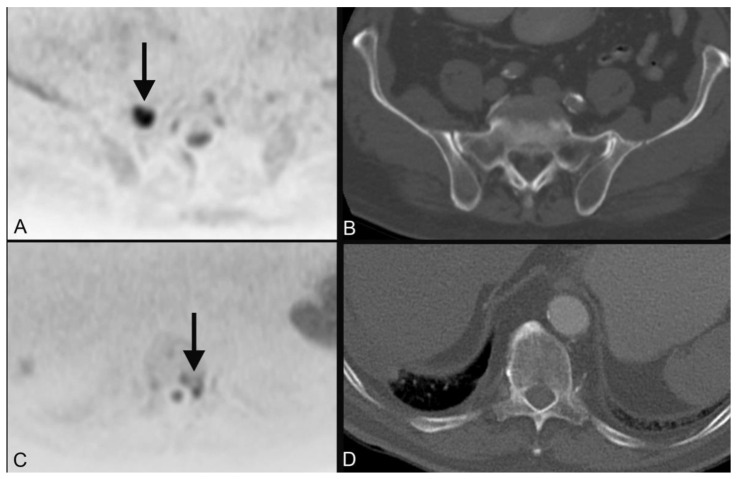
An 85-year-old male patient who was diagnosed with cancer of the right palatine tonsil (cT1 cN2b cM0) showed diffuse spotty diffusion restrictions in DWIBS of the skeletal system with the largest lesions in the right sacral mass (**A**, black arrow) and the 11th thoracic vertebrae on the left (**C,** black arrow) without correlate in the CT (**B**,**D**) and was later diagnosed with multiple myeloma.

**Figure 4:**
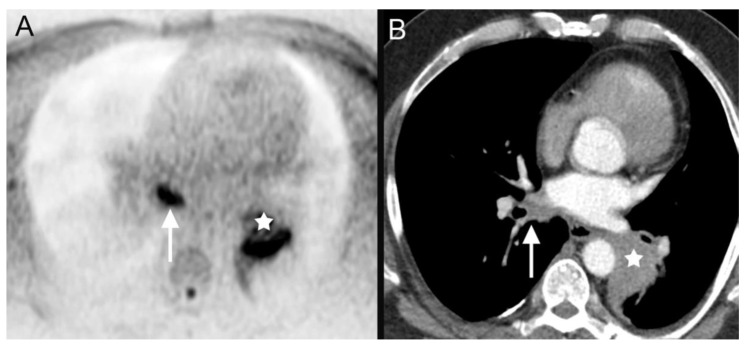
DWIBS (**A**) as well as contrast-enhanced thoracal CT (**B**) depicts the primary cancer lesion (**A**,**B**: white star) as well as the nodal metastases (**A**,**B**: white arrows) in a 60-year-old male patient who was diagnosed with a non-small cell lung cancer (cT2b cN3 cM0) in the diagnostic work-up of clinically suspected head and neck carcinoma.

**Figure 5:**
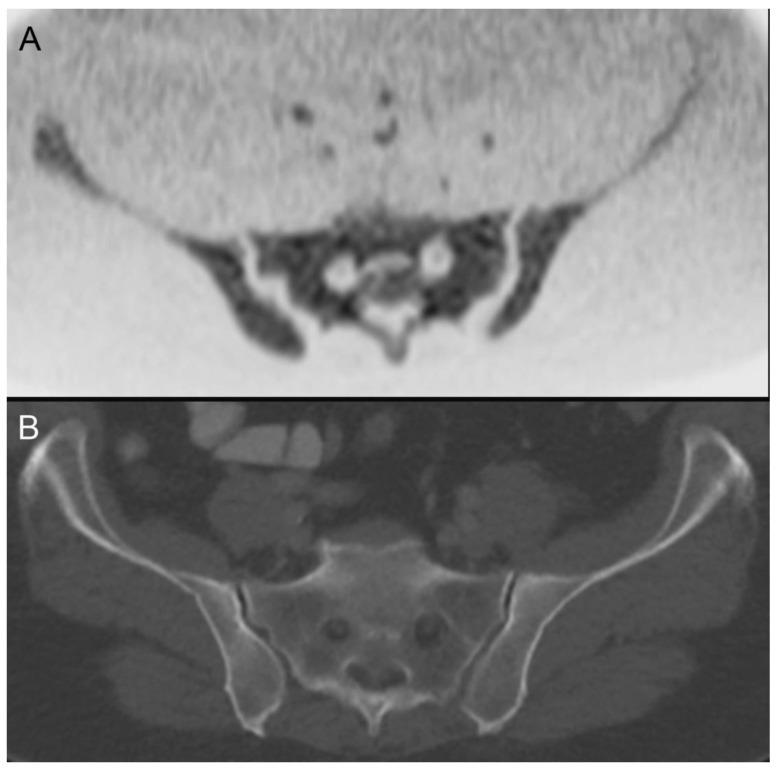
DWIBS (**A**) contrary to contrast-enhanced abdominal CT (**B**) reveals diffuse diffusion restriction of the bone marrow with no abnormalities in CT, which lead to the diagnosis of acute myeloid leukemia after further clinical work-up in this 76-year-old female patient.

**Table 1:** Risk factors.

Alcohol Consumption (*n*/%)	History of Smoking (*n*/%)	HPV+ (*n*/%)
13/43%	21/70%	6/20%

**Table 2:** Final diagnosis and TNM stage as well as histopathologic subtype of all included patients with clinically suspected head and neck carcinoma and cervical nodal swelling.

Patient Number	Diagnosis	tnm Stage	Histopathology
**1**	tonsil carcinoma	pT1 cN2b cM0	squamous cell carcinoma
**2**	laryngeal carcinoma	cT2 cN1 cM0	squamous cell carcinoma
**3**	tonsil carcinoma	pT1 pN3b cM0	squamous cell carcinoma
**4**	bronchial carcinoma	not applicable	adenocarcinoma
**5**	CUP syndrome	pTx pN+ cM0	squamous cell carcinoma
**6**	CUP syndrome	pTx pN+ cM0	squamous cell carcinoma
**7**	parotid cancer	pT3 pN2b cM0	squamous cell carcinoma
**8**	laryngeal carcinoma	cT4a cN2a cM0	squamous cell carcinoma
**9**	tonsil carcinoma	pT1 pN2b cM0	squamous cell carcinoma
**10**	hypopharyngeal carcinoma	cT4a cN2b cM0	squamous cell carcinoma
**11**	tonsil carcinoma	cT4a cN2c cM0	squamous cell carcinoma
**12**	CUP syndrome	pTx pN+ cM0	squamous cell carcinoma
**13**	tongue base carcinoma	pT1 pN2b cM0	squamous cell carcinoma
**14**	CUP syndrome	pTx pN+ cM0	squamous cell carcinoma
**15**	tonsil carcinoma	cT2 cN2c cM0	squamous cell carcinoma
**16**	lymphoma	not applicable	anaplastic large cell lymphoma
**17**	tonsil carcinoma	pT1 pN2b cM0	squamous cell carcinoma
**18**	leukemia	not applicable	acute myeloid leukemia
**19**	bronchial carcinoma	not applicable	squamous cell carcinoma
**20**	hypopharyngeal carcinoma	pT2 pN3b cM1	squamous cell carcinoma
**21**	tongue base carcinoma	pT1 cN2b cM0	squamous cell carcinoma
**22**	metastatic melanoma	not applicable	malignant melanoma
**23**	CUP syndrome	pTx pN+ cM0	squamous cell carcinoma
**24**	hypopharyngeal carcinoma	cT3 cN2b cM0	squamous cell carcinoma
**25**	thyroid carcinoma	pT4a pN1b cM1	follicular thyroid carcinoma
**26**	lymphoma	not applicable	follicular b-cell lymphoma
**27**	epiglottic carcinoma	cT1 cN2b cM0	squamous cell carcinoma
**28**	hypopharyngeal carcinoma (Figure 2)	pT2 pN2b cM0	squamous cell carcinoma
**29**	hypopharyngeal carcinoma	cT4a cN2c cM0	squamous cell carcinoma
**30**	sinunasal carcinoma	cT2 cN2b cM0	neuroendocrine carcinoma

**Table 3:** Additional diagnosis made with computed tomography (CT) and/or whole-body DWIBS MRI in the routine work-up of pretherapeutic staging.

Patient Number	Additional Diagnosis CT Only	Additional Diagnosis DWIBS Only	Additional Diagnosis in Both Modalities
1		plasmocytoma (Figure 3)	
4			bronchial carcinoma (Figure 4)
10		esophageal cancer	
16			breast lymphoma
18		leukemia (Figure 5)	
19	bronchial carcinoma		
20			single liver metastases
22		several metastases of malignant melanoma	
25		several metastases of thyroid cancer	

**Table 4:** Excerpt of the further therapeutic course of the 30 included patients based on the decision of the institutional multidisciplinary tumor conference.

Patient Number	Diagnosis	Therapeutic Decision (Multidisciplinary Tumor Conference)
**1**	tonsil carcinoma	surgical resection, adjuvant chemoradiotherapy
**2**	laryngeal carcinoma	primary chemoradiotherapy
**3**	tonsil carcinoma	surgical resection, adjuvant chemoradiotherapy
**4**	bronchial carcinoma	systemic chemotherapy
**5**	CUP syndrome	primary chemoradiotherapy
**6**	CUP syndrome	primary chemoradiotherapy
**7**	parotid cancer	surgical resection only as patient denies chemotherapy as well as radiotherapy
**8**	laryngeal carcinoma	primary chemoradiotherapy as patient denies tracheotomy
**9**	tonsil carcinoma	surgical resection, adjuvant chemoradiotherapy
**10**	hypopharyngeal carcinoma	palliative radiotherapy
**11**	tonsil carcinoma	best supportive care
**12**	CUP syndrome	primary chemoradiotherapy
**13**	tongue base carcinoma	surgical resection, adjuvant chemoradiotherapy
**14**	CUP syndrome	primary chemoradiotherapy
**15**	tonsil carcinoma	primary chemoradiotherapy
**16**	lymphoma	chemoradiotherapy
**17**	tonsil carcinoma	surgical resection, adjuvant chemoradiotherapy
**18**	leukemia	chemotherapy
**19**	bronchial carcinoma	systemic chemotherapy
**20**	hypopharyngeal carcinoma	palliative chemotherapy
**21**	tongue base carcinoma	surgical resection, adjuvant chemoradiotherapy
**22**	metastatic melanoma	immunotherapy
**23**	CUP syndrome	primary chemoradiotherapy
**24**	hypopharyngeal carcinoma	primary chemoradiotherapy
**25**	thyroid carcinoma	surgical resection and radio-iodine treatment
**26**	lymphoma	chemoradiotherapy
**27**	epiglottic carcinoma	surgical resection, adjuvant chemoradiotherapy
**28**	hypopharyngeal carcinoma (Figure 2)	primary chemoradiotherapy
**29**	hypopharyngeal carcinoma	best supportive care
**30**	sinunasal carcinoma	neoadjuvant chemoradiotherapy, surgical resection

## Data Availability

Data is available from the authors on request.

## Abbreviations

CE-CT	contrast-enhanced computed tomography
CI95	95% Confidence interval
CT	computed tomography
CUP	cancer of unknown primary
DWIBS	diffusion-weighted imaging with background suppression
FN	false negative
FNR	false negative rate
FP	false positive
FPR	false positive rate
LR+	positive likelihood ratio
LR−	negative likelihood ratio
N	negative
NPV	negative prediction value
P	positive
PPV	positive prediction value
SD	standard deviation
TN	true negative
TNR	true negative rate
TP	true positive
TPR	true positive rate
